# Children's Hospital Management in the COVID-19 Era: The Reorganization of a Tertiary Care Pediatric Emergency Department in Northern Italy

**DOI:** 10.3389/fped.2020.594831

**Published:** 2020-11-19

**Authors:** Daniele Donà, Susanna Masiero, Paola Costenaro, Marco Todeschini Premuda, Sara Rossin, Giorgio Perilongo, Anna M. Saieva, Carlo Giaquinto, Liviana Da Dalt

**Affiliations:** ^1^Division of Pediatric Infectious Diseases, Department of Women's and Children's Health, University Hospital of Padua, Padua, Italy; ^2^Pediatric Emergency Department, Department of Women's and Children's Health, University Hospital of Padua, Padua, Italy; ^3^Department of Women's and Children's Health, University Hospital of Padua, Padua, Italy; ^4^Medical Staff Affairs, University Hospital of Padua, Padua, Italy

**Keywords:** COVID-19, children, pediatric department, hospital, clinical pathway

## Abstract

In the Veneto Region, an exponential spread of patients affected by 2019 novel Coronavirus disease (COVID-19) has been observed after February 21st. Since then, we have been evaluating children suspected or confirmed for SARS-CoV-2 infection. A protocol for pediatric hospital reorganization and children management has been developed, since the beginning of the epidemic. A pre-triage area has been created at the immediate entrance of the pediatric emergency room, for all uncritical pediatric patients. According to the epidemiologic and clinical risk factors, all children/adolescents have been addressing to one of the four different pathways created. The strict application of this protocol has been leading to quickly identification, isolation, and management of all positive children, preventing SARS-CoV-2 intrahospital spread.

## Introduction

The global health crisis of SARS-CoV-2 pandemic is changing the world (1, 2). In the Veneto Region, an exponential spread of patients affected by 2019 novel Coronavirus disease (COVID-19) has been observed since February 21st, the day of the first COVID-19 positive adult admitted to the University Hospital of Padua. Since then, several actions have been immediately taken to ensure a prompt recognition of children suspected or confirmed for SARS-CoV-2 infection and to guarantee both urgent care to COVID-19 infected children and the safety of all healthcare workers and other non-infected children of the Pediatric Department of the University Hospital of Padua (3).

Case definition for pediatric suspected COVID-19 was adapted from the definition provided by the Italian Ministry of Health, therefore a *suspected case* was defined as a child/adolescent with **fever (TC>** **37.5 C axillary)** and/or **respiratory symptoms (rhinitis, cough, and dyspnea)** and/or **gastrointestinal symptoms (vomiting, diarrhea)** with or without close contact with a probable or confirmed case of COVID-19, within the previous 14 days (4, 5). Traveling in high risk areas was no longer considered as epidemiologic risk factor, because Italy was declared “red zone” at the beginning of March. *COVID-19 confirmed cases* were those with a **positive nasopharyngeal swab test for SARS-CoV-2**, detected by qualitative polymerase-chain reaction (PCR) (4).

This protocol was the result of all our daily efforts to merge scientific evidences that were available with clinical and organizational issues faced within the first month of coexistence with COVID-19 (6–9).

The aim of this paper is to share our experience in order to support other pediatricians in different settings in dealing with the structural reorganization of Pediatric Departments facing with COVID-19 pandemic.

### Setting

In March 2020, this hospital reorganization was, firstly, set up in the PED of the Department of Woman's and Child's Health at Padua University Hospital and it was subsequently promoted to all the Pediatric departments of Veneto Region.

Our Children's Hospital provides primary and secondary care for a metropolitan area of 350,000 people (45,000 younger than 15 years) and tertiary care for a regional and extra-regional population, with ~26,000 PED visits per year and an overall hospital admission rate from PED of around 7 out of 100 visits.

### Policy Options and Implications

Several operational steps have been set up in order to reorganize the PED and the Pediatric Units.

## Reorganization of the Emergency Room (ER) (Figure 1)

### ER Presentation of Uncritical Patient

A pre-triage area has to be created at the immediate entrance of every pediatric or general emergency room (ER), to evaluate all uncritical pediatric patients that **autonomously** come from home. The pre-triage evaluation is crucial to promptly identify patients at risk of COVID-19, before hospital admission. The evaluation must be performed by trained healthcare workers with adequate personal protective equipment (PPE) and a surgical mask must be given to the child and his/her caregiver.

**Figure 1:**
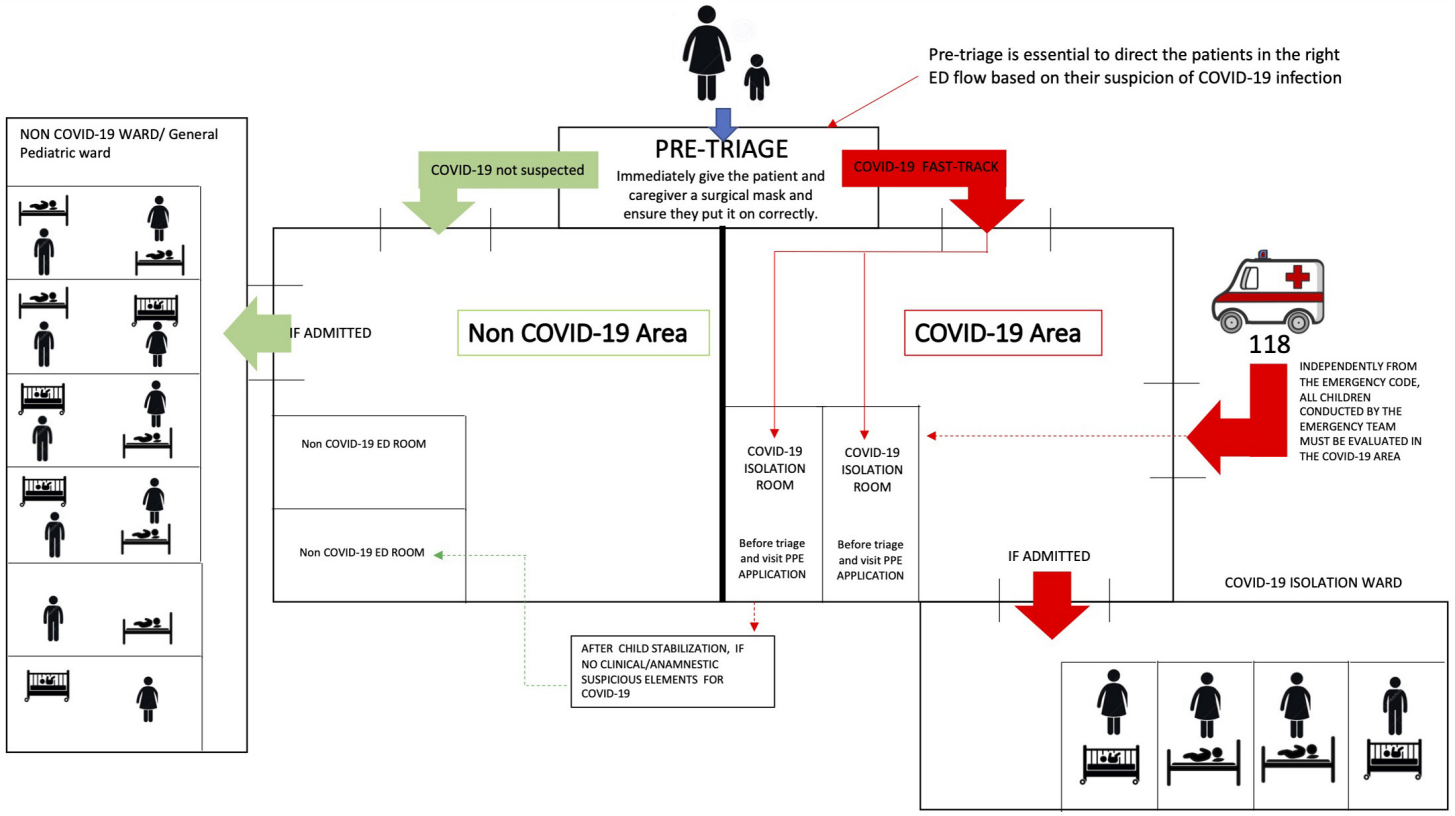
Pediatric Emergency Department reorganization in the COVID-19 era.

Following the pre-triage, two different areas have to be created in order to separately direct patients and their caregivers to specifically dedicated areas, such as:

- **Suspected COVD-19 child/adolescent area (COVID-19 Area)**, ideally provided with at least one negative pressure isolation room.
- **Not suspected COVID-19 child/adolescent area (Not COVID-19 Area)**.

The two Areas must be physically separated, with dedicated healthcare workers in order to avoid grouping and contamination among suspected/confirmed cases and those who are not.

In the **COVID-19 Area**, a dedicated waiting room have to be set up for the child/adolescent and his/her caregiver, with recommendation of wearing surgical masks and keeping at least 1 m of distance from other patients. Whenever these conditions are not feasible, the child/adolescent and his/her caregiver have to be isolated in the evaluation room.

### ER Presentation of Critically Ill Patient

All patients urgently conducted to the emergency room by an emergency team have to be directly sent to the isolation room of **COVID-19 Area**, being assisted by healthcare workers equipped with adequate PPE, since a pre-triage assessment is not performed.

As soon as the child/adolescent is defined as stable, he/she have to be transferred to the **Not COVID-19 Area** if absence of any epidemiologic risks and/or signs and symptoms suspicious of COVID-19.

## Reorganization of Pediatric Units

Every hospital with an ER must arrange rooms for suspected or confirmed COVID-19 pediatric patients, separated from the non-COVID-19 patients ward.

Whenever available, the child/adolescent should be placed in a negative pressured room or, if it is not possible, in a single-bed room with its own bathroom setting up all precautions needed for respiratory diseases, such as standard precautions and adjunctive protection devices for agents transmittable by droplets and/or through aerosol and/or contact. All patients with confirmed COVID-19 should be assisted by dedicated staff. The patient's caregiver should always wear a surgical mask and should not exit the room. Indeed, visitors have not to be allowed. Meals have to be served with disposable cutlery to both the patient and his/her caregiver.

In case both the child/adolescent and his/her caregiver are affected by COVID-19 infection, it would be reasonable to hospitalize them together in a dedicated room of the pediatric ward, to guarantee the family unity, if the caregiver is asymptomatic or has mild symptoms (which normally do not required hospitalization).

If parents/caregivers require hospitalization due to clinical conditions, the child and caregivers have to be referred to an Infectious Diseases ward and a dedicated pediatric consultant should be activated.

In case of a critically ill child/adolescent with confirmed COVID-19, the patient should be immediately transferred to the referral hospitals with COVID-19 dedicated pediatric intensive care units.

## Pathways for Child/Adolescent With Suspected or Confirmed Infection by SARS-CoV-2

**Figure 2:**
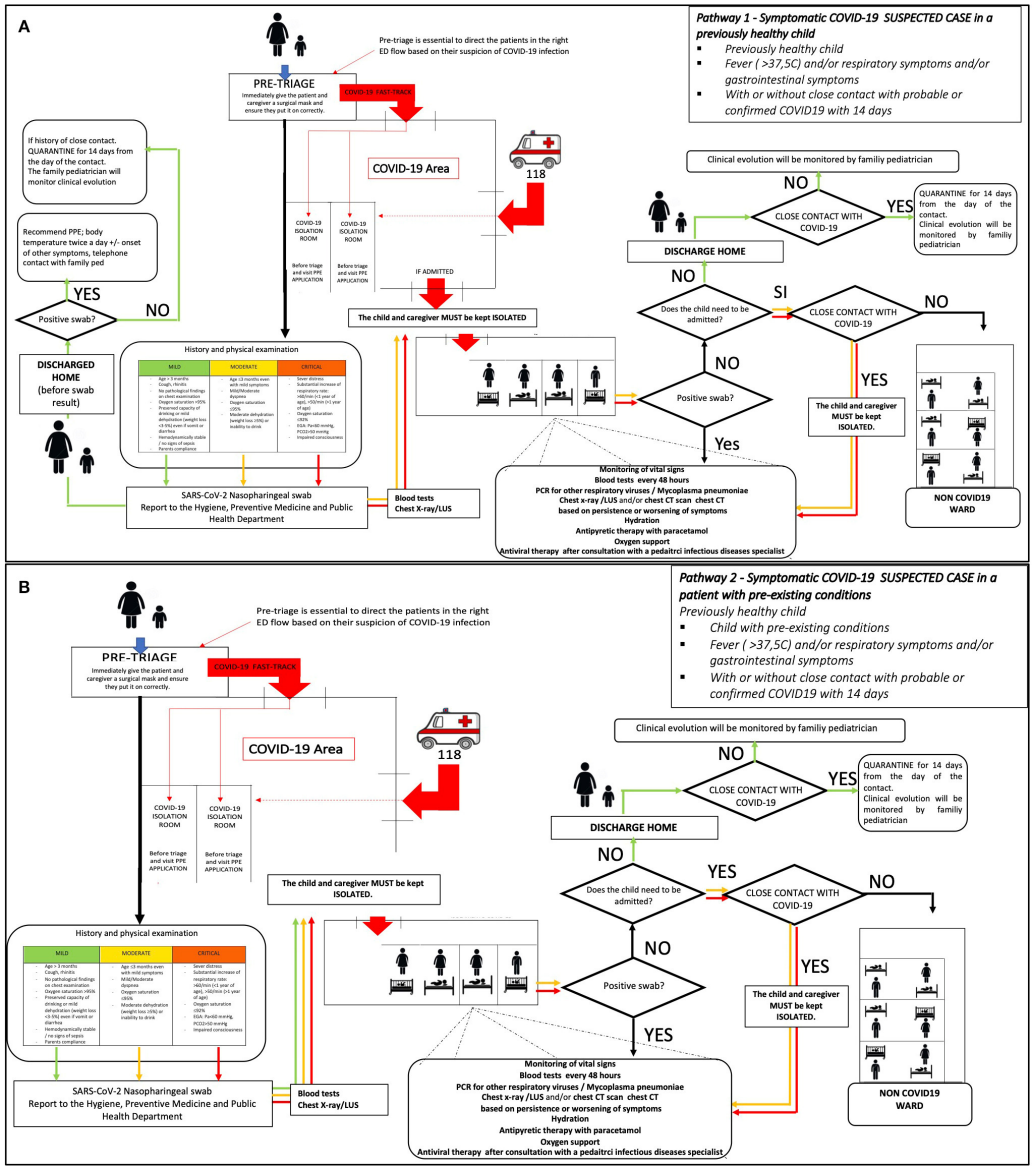
**(A)** Pathway 1—management of a previously healthy child/adolescent, suspected, or probable case of COVID-19. **(B)** Pathway 2—management of a child/adolescent with chronic disease /immunosuppression suspected or probable case of COVID-19.

After being evaluated at pre-triage, patients should be stratified according to clinical characteristics, past history including concomitant co-morbidities, and epidemiological risk of COVID-19. The following four **different pathways** have been defined:

***Pathway 1—Symptomatic COVID-19 SUSPECTED CASE in a previously healthy child (******Figure 2A******)***

- *Previously healthy child*.
- *Fever (*>*37.5*°*C) and/or respiratory symptoms and/or gastrointestinal symptoms*.
- *With or without close contact with probable or confirmed COVID19 cases in the previous 14 days*.

***Pathway 2—Symptomatic COVID-19 SUSPECTED CASE in a patient with pre-existing conditions (******Figure 2B******)***

- *Child with pre-existing conditions*.
- *Fever (*>*37.5*°*C) and/or respiratory symptoms and/or gastrointestinal symptoms*.
- *With or without close contact with probable or confirmed COVID19 cases in the previous 14 days*.

**Figure 3:**
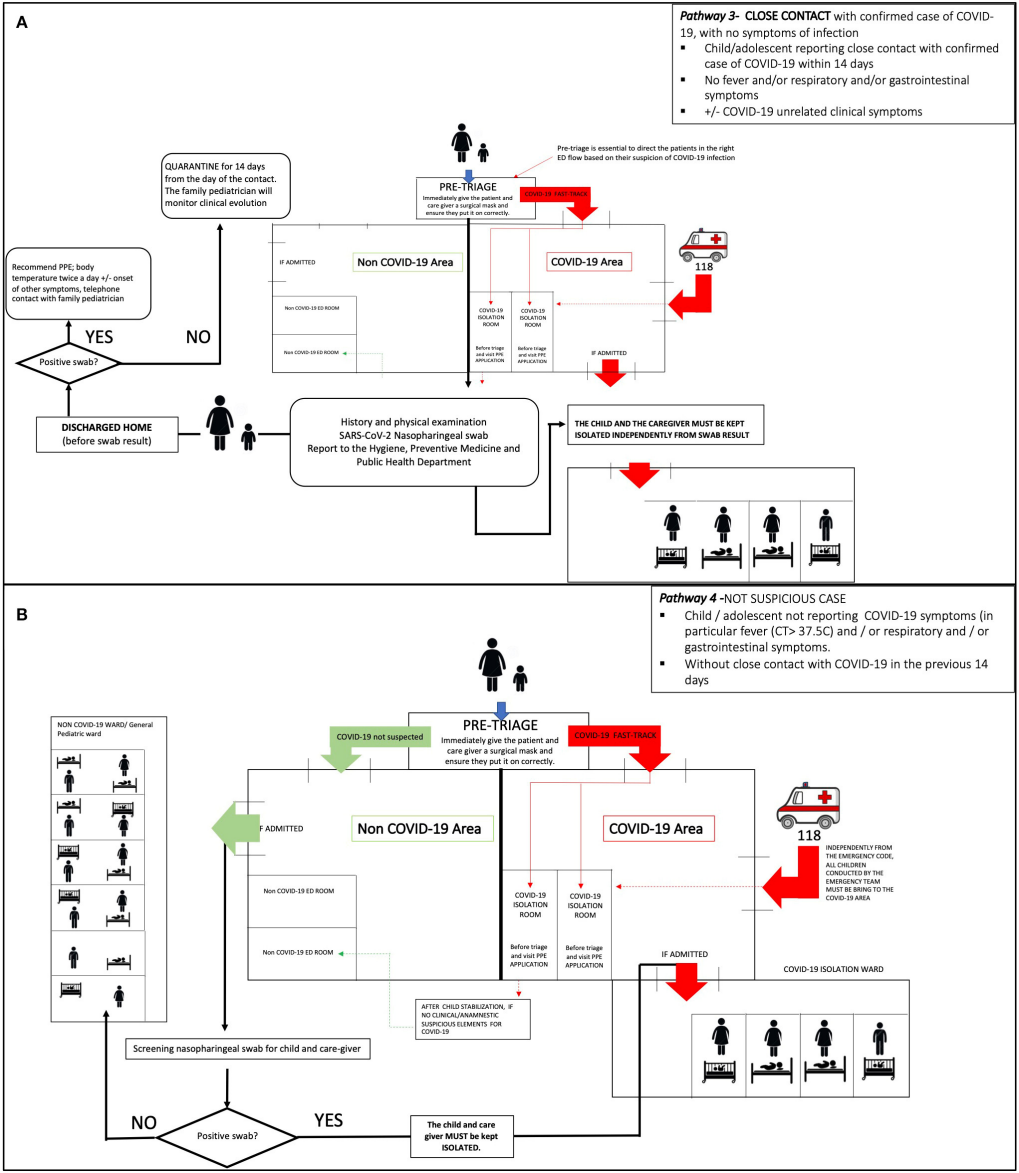
**(A)** Pathway 3—management of an asymptomatic child/adolescent with confirmed “close contact.” **(B)** Pathway 4—management of a child/adolescent NOT SUSPECTED for COVID-19.

***Pathway 3—CLOSE CONTACT with confirmed case of COVID-19, with no symptoms of*** ***infection (******Figure 3A******)***

- *Child/adolescent reporting close contact with a confirmed case of COVID-19 within 14 days*.
- *No fever and/or respiratory and/or gastrointestinal symptoms*.
- ± *COVID-19 unrelated clinical symptoms*.

***Pathway 4—NOT SUSPICIOUS CASE (******Figure 3B******)***

- *Child/adolescent not reporting COVID-19 symptoms in particular fever (CT* > *37.5*°*C) and/or respiratory and/or gastrointestinal symptoms*.
- *Without close contact with COVID-19 cases in the previous 14 days*.

### Pathway 1—“*Symptomatic COVID-19 Suspected Case in a Previously Healthy Child” (**Figure 2A**)*

Patients identified at pre-triage as “COVID-19 SUSPECTED CASE” and not affected by any chronic condition should be sent to the evaluation room of **COVID-19 Area**, after wearing a surgical mask.

After being evaluated for family history and past medical history, including immunizations and allergies, the child and his/her caregiver should be clinically evaluated and classified as “mild—moderate or critical” according to the following proposed classification (Table 1). This score was formulated on the basis of available evidence, at the time of protocol set up (6–9). To define moderate/critical clinical syndrome **just one** of the Table 1 criteria is need.

**Table 1:** Clinical classification for SARS-CoV-2 infected children.

**Mild**	**Moderate**	**Critical**
- Age > 3 months - Cough, rhinitis - No pathological findings on chest examination - Oxygen saturation >95% - Preserved capacity of drinking or mild dehydration (weight loss <3–5%) even if vomit or diarrhea Hemodynamically stable/no signs of sepsis - Parents compliance	- Age ≤3 months even with mild symptoms - Mild/Moderate dyspnea - Oxygen saturation ≤95% - Moderate dehydration (weight loss ≥5%) or inability to drink	- Sever distress - Substantial increase of respiratory rate: >60/min (<1 year of age), >50/min (>1 year of age) - Oxygen saturation ≤92% - EGA: Pa <60 mmHg, PCO2>50 mmHg - Impaired consciousness

After clinical evaluation, *all children/adolescents have to be tested with:*

- Nasopharyngeal swab for SARS-CoV-2.
- Rectal swab for SARS-CoV-2 (whenever possible).

We strongly suggest testing also the caregiver with a nasopharyngeal swab for SARS-CoV-2 since familiar clusters are frequent.

The following further tests have to be performed in case of moderate/critical clinical presentation:

- **Chest X-Ray** and/or **lung ultra-sound (LUS)** and/or **chest CT scan**, using portable diagnostic tools (to avoid any transfer of infected patients to other areas).
- **Blood tests:** full blood count, renal and liver function, glucose, CRP, PCT, hemogasanalysis, urinalysis, if septic myocardial enzymes, PT, PTT, fibrinogen, FDP, LDH, ferritin, lactate.

The pediatrician will decide whether to discharge or admit the child/adolescent according to **clinical criteria** (while waiting for tests results).

If the patient has mild symptoms can be discharged before swabs result, with strict indication for home isolation while waiting for the results. The results have to be reported to the caregiver or to the pediatrician within 24–48 h. Children and their families have to be referred to the family pediatrician (FP) and/or general practitioner (GP), for further follow-up.

Based on swab's results, the child home-based management has to be organized as follows:

- In case of a *positive result* for COVID-19, the child discharged from the ER should undergo home isolation and caregivers must apply contact or droplets precaution (if negatives) to prevent viral transmission. Body temperature should be checked twice a day as the onset of respiratory and gastrointestinal symptoms. In case of worsening of symptoms, they should be further referred to the ER. Home isolation have to be kept for at least 14 days, at the end of that period two consecutive nasopharyngeal swab for SARS-CoV-2 have to be performed: isolation should end only if both swabs are negative. Active surveillance for 14 days from last contact with COVID-19 have to be applied to all “close contacts.”
- In case of a *negative swab*, a 14-day home isolation should be recommended (from the last day of contact) to al close contacts with a confirmed COVID-19 case. The clinical evolution will be monitored by the GP.

All suspected/confirmed COVID-19 cases classified as moderate/severe have to be hospitalized in a dedicated area, regardless of COVID-19 swab pending results. Critically ill children/adolescents with suspected/confirmed COVID-19 should be stabilized and referred to hospitals with COVID-19 dedicated pediatric intensive care units, particularly if the patient is younger than 1 year of age, due to the higher risk of worsening conditions.

The further management of admitted children/adolescents will change according to the results of nasopharyngeal swab test:

- In case of *positive result*, the patient should be kept hospitalized in the confirmed/suspected COVID-19 dedicated ward (regular or ICU). Please refer to section 4-Management of COVID-19 positive hospitalized patient.
- In case of *negative result*, the child or adolescent should be transferred to another non-COVID-19 ward, only in the absence of epidemiological risk factors. In case of history of close contact, the patient should be kept isolated in the COVID-19 ward.

### Pathway 2—“*Symptomatic* Suspected/Probable” COVID-19 Case in an Immunocompromised Patient/Patient With Chronic Disease (Figure 2B)

Patients identified at pre-triage as “COVID-19 SUSPECTED CASE” should be sent to the evaluation room of **COVID-19 Area**, after wearing a surgical mask. Please see section Pathway 1—“*Symptomatic COVID-19 Suspected Case in a Previously Healthy Child” (**Figure 2A**)* for the clinical evaluation.

All symptomatic children/adolescents with suspected COVID-19 and with concomitant immunosuppression/chronic disease have to be admitted to the hospital for observation.

The further management of admitted children/adolescents changes according to the results of nasopharyngeal swab test:

- in case of a positive result, the patient should be hospitalized in a confirmed/suspected COVID-19 dedicated ward (pediatric ward or PICU). Please refer to section Management of COVID-19 positive hospitalized patient. Home isolation with active surveillance should be applied to all “close contacts,” for 14 days from patient's last contact.
- in case of *negative result*, the child or adolescent should be transferred to another non-COVID-19 ward, only in the absence of epidemiological risk factors. In case of history of close contact, the patient should be kept isolated in the COVID-19 ward. If clinical conditions do not require hospitalization, the patient should be discharged recommending home isolation and active surveillance only in case of a referred COVID-19 close contact. Clinical evolution should be monitored by the GP.

### Pathway 3—Close Contact With a Confirmed COVID-19 Case, Asymptomatic for COVID-19 Symptoms (e.g., ER Admission for Different Clinical Issues) (Figure 3A)

Patients identified at pre-triage as “COVID-19 SUSPECTED CASE” and not affected by any chronic condition should be sent to the evaluation room of **COVID-19 Area**, after wearing a surgical mask. After COVID-19 test has been performed, the pediatrician should evaluate whether to discharge or hospitalize the child/adolescent according to disease severity.

If the child/adolescent is clinically stable, he/she should be discharged before the COVID-19 test result and home isolation have to be recommended until the availability of test result, that should be reported to the caregiver or to GP within 24–48 h. In case of children/adolescents with clinical course requiring hospitalization, they should be referred to a COVID-19 dedicated ward.

### Pathway 4—Non-suspected Case (Figure 3B)

The healthcare worker of pre-triage has to address the patient and his caregiver to the triage in the **NOT COVID-19 Area**. They will be evaluated according to the disease that drove them to the ER.

The nasopharyngeal swab screening has to be performed to the child and caregiver only if hospitalization is needed, to prevent in-hospital virus spread from asymptomatic children.

## Management of COVID-19 Positive Hospitalized Patient

### Monitoring

- Vital parameters: Temperature, heart rate, respiratory rate, blood pressure, and oxygen saturation.
- Blood tests.
- Viral PCR for other respiratory viruses and *Mycoplasma pneumoniae*.
- Chest x-ray or CT scan if respiratory symptoms persist or worsen.

### Supportive Care

- Hydration with appropriate caloric and electrolytic intakes.
- Antipyretic drugs: paracetamol as first line and ibuprofen as second line (there is no clinical evidence that defines a correlation between ibuprofen and the worsening of clinical conditions due to evolution of COVID-19. As the national guidelines and EMA suggest, patients may continue the use of NSAIDs (10).

### Steroid Therapy

The use of corticosteroids is not contraindicated for concomitant treatment of other underlying diseases (e.g., asthma) if benefits are greater than the risks. In patients with chronic use of corticosteroids any modification has to be arranged with the referent specialist/consultant.

### Respiratory Support

- Moderate cases: oxygen mask with a target of oxygen saturation >95%.
- Oxygen with HFNC if the target is not achieved with the mask. In this case, we do suggest contacting the nearest center with a dedicated PICU.
- Severe cases: CPAP, NIV with early intubation, and mechanical ventilation.

### Target Therapy

Because of poor evidence, any antiviral and/or immunomodulatory therapy should be considered case by case and defined after Pediatric Infectious Diseases consultation. For each specific case, a multidisciplinary team that includes a Pediatric Infectious Diseases consultant must review the last evidences on antiviral and/or immunomodulant treatments for Covid-19, in order to consider if the patient can be included in any trial and/or if he/she can apply for the compassionate use of any further treatment, including the use of Remdesivir, a nucleotide analog prodrug that inhibits viral RNA polymerases of SARS-CoV-2, the use of hyperimmune plasma from patients recovered from Covid-19 and/or the use of any immunomodulant treatment including targeted anti-inflammatory products such as interleukin inhibitors, interferons, kinase inhibitors, and others (11–13).

Confirmed COVID-19 hospitalized patients must be kept isolated till clinical recovery. This is defined by 48-72 of apyrexia AND respiratory/gastrointestinal symptoms resolution. When discharged the patents should continue isolation till two negative swabs results 24 h apart.

### Actionable Recommendations

During this first month of COVID-19 emergency, our Department faced mostly organizational issues in children management. Although during the lockdown period (6th March−4th May) the ER utilization had a significant reduction (−75%) a series of critical issues arose as the need of different areas and isolation rooms for infected patients and the need of clear pathways for the management of all patients according to different epidemiologic and/or clinical characteristics. Between the 6th March and 4th May, 1,291 patients were evaluated and 416 (32.2%) were tested for SARS-CoV-2. Two-hundred and fifty-nine children (20.1%) sought medical evaluation for fever and/or respiratory or gastrointestinal symptoms (pathways 1 and 2). All patients received the SARS-CoV-2 test and for 6/416 this turned positive. All close contact with a confirmed COVID-19 case admitted to our ER (24/416) were tested and 4/24 (16.7%) were found positive (pathway 3). Most of the children (83.1%) were referred to our ER for non-COVID-19 related problems. As per pathway 4, SARS-CoV-2 tests were performed only in case of hospital admission: 92/416 children were tested, all with negative results (Table 2).

**Table 2:** Padua PED 5-month experience stratified by four different pathways.

	**Phase 1 (6 March−4 May 2020)**			**Phase 2 (5 May−31 July 2020)**		
	**PED evaluations** ***n*** **=** **1,291**			**PED evaluations** ***n*** **=** **3,593**		
	**SARS-CoV-2 nasopharyngeal swab performed**		**SARS-CoV-2 nasopharyngeal swab not performed**	**SARS-CoV-2 nasopharyngeal swab tests performed**		**SARS-CoV-2 nasopharyngeal swab not performed**
	**416**		**875**	**768**		**2,825**
	**Positive**	**Negative**		**Positive**	**Negative**	
**Pathway #1**— SUSPECTED/PROBABLE COVID-19 case in a previously healthy patient	6	253	0	0	587	0
**Pathway #2**— SUSPECTED/PROBABLE COVID-19 case in an immunocompromised patient/patient with chronic disease	1	40	0	0	45	0
**Pathway #3**— CLOSE CONTACT with confirmed COVID-19 case. Asymptomatic for COVID-19 symptoms	4	20	0	5	14	0
**Pathway #4**—NON-COVID-19 SUSPECTED case	0	92	875	0	117	2,825
Total	11	405	875	5	763	2,825

After the lockdown period, with the gradual return to the usual PED workload, this protocol became even more crucial to guarantee the safety of all healthcare workers and other non-infected children admitted to the hospital. Three-thousand and ninety-three patients were evaluated and 768 (21.4%) were tested for SARS-CoV-2. Despite the increase in ER evaluations for fever and/or respiratory or gastrointestinal symptoms (587 vs 259 visits), none of these resulted positive for SARs-CoV-2. On the other hand, 5/19 (26.3%) children close contact with a confirmed COVID-19 case were found positive. One-hundred and seventeen were referred to our ER for non-COVID-19 related problems (Table 2).

Moreover, starting from March 4th, all health care workers have been screened every 10 days for SARS-CoV-2 through a nasopharyngeal swab. No cases of intra-department infection were documented among the healthcare workers and other admitted patients since all the preventive procedures described above were implemented.

Based on this 5-month experience, the following widely generalizable recommendations have been identified to face with COVID-19 pandemic:

- A pre-triage assessment has to be organized at the immediate entrance of the ER, in order to promptly guarantee patients identification and to address them to the most appropriate pathway.
- Clear and differentiated pathways have to be created for the management of all pediatric patients, according to different epidemiologic and/or clinical characteristics.
- Different areas and isolation rooms have to be arranged in order to separate SARS-CoV-2 infected and/or suspected patients from patients unlikely to be affected by COVID-19.
- Education of health care workers on infection prevention and control measures and on COVID-19 related operational procedures and standards has to be considered as a priority, in order to minimize the risk of in-hospital spread of SARS-CoV-2 infection.

## Conclusions

In-hospital spread of SARS-CoV-2 infection can be avoided through the implementation of clear and practical procedures aimed at promptly recognize and address pediatric patients and their caregivers, soon after being admitted to the pediatric emergency room. These measures, including a pre-triage area and specific and well-differentiated pathways based on patient's epidemiologic and clinical risk factors, are simple to implement and extremely important to quickly identify, isolate and manage all positive children, therefore must be planned and realized. We believe that our experience may be transferred to other similar settings, as a support for the implementation of hospital-based protocols aimed at containing the spread of SARS-CoV-2 infection, both at local and global levels.

## Author Contributions

Manuscript conceptualization: DD, SM, and LD. Data Curation: DD, MT, SR, and PC. Writing—Original draft preparation: DD, SM, PC, MT, and SR. Writing—review and editing: CG, GP, AS, and LD. Visualization and supervision: DD and LD. Each author has approved the submitted version. All authors have made substantial contributions to the conception and design of the protocol.

## Conflict of Interest

The authors declare that the research was conducted in the absence of any commercial or financial relationships that could be construed as a potential conflict of interest.

## References

[1] WHO Coronavirus Disease (COVID-2019) Situation Report 196 (2020).

[2] Callaway E Time to use the p-word? Coronavirus enter dangerous new phase. Nature. (2020) 579:12 10.1038/d41586-020-00551-1 33623145

[3] Remuzzi A Remuzzi G. COVID-19 and Italy: what next? Lancet. (2020) 395:1225–8. 10.1016/S0140-6736(20)30627-9 32178769 PMC7102589

[4] COVID-19 Case Definition. Italian Ministry of Health Available online at: http://www.trovanorme.salute.gov.it/norme/renderNormsanPdf?anno=2020&codLeg=73669&parte=1%20&serie=null

[5] Donà D Minotti C Costenaro P Da Dalt L Giaquinto C. Fecal-oral transmission of SARS-CoV-2 in children: is it time to change our approach?. Pediatr Infect Dis J. (2020) 39:e133–e4. 10.1097/INF.0000000000002704 32304466 PMC7279053

[6] Parri N Lenge M Buonsenso D. Coronavirus Infection in Pediatric Emergency Departments (CONFIDENCE) Research Group. Children with Covid-19 in Pediatric Emergency Departments in Italy. N Engl J Med. (2020) 383:187–90. 10.1056/NEJMc2007617 32356945 PMC7206930

[7] Garazzino S Montagnani C Donà D Meini A Felici E Vergine G. Multicentre Italian study of SARS-CoV-2 infection in children and adolescents, preliminary data as at 10 April 2020. Euro Surveill. (2020) 25:2000600. 10.2807/1560-7917.ES.2020.25.18.2000600 32400362 PMC7219028

[8] Ludvigsson JF. Systematic review of COVID-19 in children show milder cases and a better prognosis than adults. Acta Paediatr. (2020) 109:1088–95. 10.1111/apa.15270 32202343 PMC7228328

[9] World Health Organization Clinical Management of Severe Acute Respiratory Infection When Novel Coronavirus (nCoV) Infection is Suspected. (2020) Available online at: https://www.who.int/publications-detail/clinical-management-of-severe-acute-respiratory-infection-when-novel-coronavirus-(ncov)-infection-is-suspected (accessed June 16, 2020).

[10] EMA European Medicine Agency (Ema). Available online at: http://www.salute.gov.it/portale/nuovocoronavirus/dettaglioNotizieNuovoCoronavirus.jsp?lingua=italiano&menu=notizie&p=dalministero&id=4264

[11] Chiotos K Hayes M Kimberlin DW Jones SB James SH Pinninti SG. Multicenter initial guidance on use of antivirals for children with COVID-19/SARS-CoV-2. J Pediatric Infect Dis Soc. (2020. 10.1093/jpids/piaa045. [Epub ahead of print]. 32318706 PMC7188128

[12] Minotti C Tirelli F Barbieri E Giaquinto C Donà D. How is immunosuppressive status affecting children and adults in SARS-CoV-2 infection? A systematic review. J Infect. (2020) 81:e61–e6. 10.1016/j.jinf.2020.04.026 32335173 PMC7179496

[13] Venturini E Montagnani C Garazzino S Donà D Pierantoni L Lo Vecchio A. Treatment of children with COVID-19: position paper of the Italian Society of Pediatric Infectious Disease. Ital J Pediatr. (2020) 46:139. 10.1186/s13052-020-00900-w 32972435 PMC7512208

